# Alternative Splicing at NAGNAG Acceptors: Simply Noise or Noise and More?

**DOI:** 10.1371/journal.pgen.0020207

**Published:** 2006-11-24

**Authors:** Michael Hiller, Karol Szafranski, Rolf Backofen, Matthias Platzer

Alternative splicing at pairs of acceptors in close proximity are one frequent cause of transcriptome complexity. In particular, acceptors with the pattern NAGNAG are widespread in several genomes [1–3]. When affecting the coding regions, alternative splicing at NAGNAGs mainly results in the insertion/deletion of one amino acid. While such subtle events are undoubtedly frequent, an important question arises: do they have functional consequences or are they simply noise tolerated by cells?

Zavolan and colleagues [3,4] suggest that these variations are the result of stochastic binding of the spliceosome at neighboring splice sites and do not discuss known functional implications. We previously found indications against a general noise assumption for NAGNAG splice events [1]: biases towards intron phase 1 and single amino acid insertions/deletions, correlation of amino acid variation and the peptide environment, enrichment of polar residues at NAGNAG exon–exon junctions, preference for protein–protein interactions and particular Pfam domains, human–mouse conservation of the intronic AG, and tissue-specific splicing at several NAGNAG acceptors. These findings indicate negative selection against NAGNAG-derived variability deleterious for certain protein regions, which agrees with the underrepresentation of NAGNAGs in coding regions detected by Zavolan and colleagues [4]. This does not rule out that variability may be advantageous for other proteins, but signs of positive selection are much harder to detect and remain to be shown.

Zavolan's finding that confirmed NAGNAGs (current mRNAs/expressed sequence tags do show alternative splicing) are not better conserved between human and mouse than unconfirmed ones may argue against functional implications. However, this result is probably biased by the unconfirmed dataset, which consists of ~60% NAGGAG whose GAG is part of the conserved exon. To avoid such a bias, we split confirmed NAGNAGs into those in which the “extra” AG is either intronic or exonic, according to the transcript annotation [1]. Interestingly, intronic but not exonic extra AGs have a significant conservation. Meanwhile, Akerman and Mandel-Gutfreund found a high conservation of the intronic flanking regions [5], typical for biologically meaningful alternative splicing [6].

The finding of Zavolan and colleagues that relative acceptor strength is predictive for confirmed and unconfirmed NAGNAGs refers to an accepted fact of splicing (for example, alternative exons have weaker splice sites than constitutive ones [7]). In tandems, the splice-site strength often determines the preferred acceptor, consistent with our earlier results (see Supplementary Notes in [1]). Thus, we agree that thermodynamic fluctuation plays an essential role during splice-site recognition at NAGNAG acceptors. This is in line with the finding that a single mutation is sufficient to convert a normal acceptor into a NAGNAG tandem, enabling alternative splicing [8]. However, this useful model is not valid for all NAGNAGs. In particular, tissue-specific regulation of alternative NAGNAG splicing challenges this model [1,9]. Overrepresented sequence motifs found in the vicinity of confirmed NAGNAGs are likely to contribute to this regulation [5].

Moreover, some protein isoforms derived by alternative splicing at NAGNAG acceptors are known to be functionally different: IGF1R, signaling [10]; DRPLA, cellular localization [9]; mouse Pax3, DNA binding [11]; and Arabidopsis thaliana U11-35K, protein binding [12]. Alternative NAGNAG splicing in the untranslated region of mouse Ggt1 affects the translational efficiency [13]. Furthermore, a NAGNAG mutation in ABCA4 is relevant for Stargardt disease 1 [14]. For clarity, we did not claim that all alternative splice events at NAGNAGs serve as protein “fine-tuning” mechanism [1,8] (as misinterpreted by [4]). In our opinion, like genetic variants, splice variants may be neutral or result in phenotypic differences. Thus, they represent just another playground of molecular evolution [15,16]. The few currently evident cases of biologically different NAGNAG-derived isoforms may represent just the tip of an iceberg.

Finally, in the context of the problem discussed here, it has to be considered that noise is important for many biological processes [17], leading to the model of “cultivated noise” [18]. For example, splicing noise at the *Drosophila Dscam* gene is used for cell individualization [19]. Although it has yet to be proven, it is tempting to speculate that noise arising by splicing at NAGNAG acceptors provides another “cultivated” stochastic mechanism.

In conclusion, it remains unknown what fraction of the more than 1,900 currently confirmed human NAGNAGs play a role in biological functions. To facilitate further experimental and bioinformatics analyses, we developed a database, TassDB (http://helios.informatik.uni-freiburg.de/TassDB), that provides information and large collections of NAGNAG acceptors.
